# Protective Effects of Crocetin against Radiation-Induced Injury in Intestinal Epithelial Cells

**DOI:** 10.1155/2020/2906053

**Published:** 2020-09-08

**Authors:** Chen Zhang, Kequan Chen, Jinghua Wang, Zhongwen Zheng, Yujun Luo, Weijie Zhou, Zewei Zhuo, Jun Liang, Weihong Sha, Hao Chen

**Affiliations:** ^1^Department of Gastroenterology, Affiliated South China Hospital, Southern Medical University (Guangdong Provincial People's Hospital), Guangzhou 510080, China; ^2^Department of Gastroenterology, Guangdong Provincial People's Hospital, Guangdong Academy of Medical Sciences, Guangzhou 510080, China; ^3^Department of Gastroenterology, The First Affiliated Hospital of Guangzhou Medical University, Guangzhou 510120, China; ^4^Department of Hematology, Guangdong Provincial People's Hospital, Guangdong Academy of Medical Sciences, Guangzhou 510080, China; ^5^Department of Critical Care Medicine, Guangdong Provincial Geriatrics Institute, Guangdong Provincial People's Hospital, Guangdong Academy of Medical Sciences, Guangzhou 510080, China

## Abstract

**Background and Aims:**

Treatment options for radiation-induced intestinal injury (RIII) are limited. Crocetin has been demonstrated to exert antioxidant, antiapoptotic, and anti-inflammatory effects on various diseases. Here, we investigate the effects of crocetin on RIII *in vitro*. *Materials and Method*. IEC-6 cells exposed to 10 Gy of radiation were treated with different doses of crocetin (0, 0.1, 1, 10, and 100 *μ*M), and cell viability was assessed by CCK-8. The levels of superoxide dismutase (SOD), catalase (CAT), glutathione peroxidase (GPx), malondialdehyde (MDA), myeloperoxidase (MPO), tumor necrosis factor-*α* (TNF-*α*), interleukin-1*β* (IL-1*β*), and interferon-*γ* (IFN-*γ*) in culture supernatants were measured using colorimetric and ELISA kits, respectively. Cellular apoptosis was evaluated by Annexin V/PI double staining.

**Results:**

Crocetin dose-dependently improved the survival of irradiated IEC-6 cells with the optimal dose of 10 *μ*M, as indicated by the reduction of cellular apoptosis, decreased levels of MDA, MPO, and proinflammatory cytokines (TNF-*α*, IL-1*β*, and IFN-*γ*), and increased activities of antioxidative enzymes (SOD, CAT, and GPx).

**Conclusion:**

Our findings demonstrated that crocetin alleviated radiation-induced injury in intestinal epithelial cells, offering a promising agent for radioprotection.

## 1. Introduction

Radiation-induced intestinal injury (RIII) is a common complication of radiation therapy in patients with abdominal or pelvic malignancies, which seriously affects the quality of life and even leads to substantial mortality [1, 2]. Exposure of the small intestine to radiation may produce a large amount of free radicals and epithelial cell apoptosis, which cause impaired barrier function, followed by inflammatory response and even septicemia [3, 4]. Although RIII seriously affects the efficacy of abdominopelvic radiotherapy, there are no therapeutic agents available to attenuate the intestinal toxicity of radiation [5].

Radioprotectors targeting oxidative damage and inflammatory reaction have been studied for decades with limited success, because of either the limited protective effect or inevitable toxicity [6]. In addition, previous studies have showed that some radioprotective agents had the risk of tumorigenesis, hampering their clinical application [6, 7].

Crocetin, an active constituent of saffron (*Crocus sativus L*) stigma, belongs to the large family of *carotenoids* [8]. Accumulated evidences have demonstrated that crocetin exerted beneficial effects on injured tissue [9, 10] and tumor cells [11, 12]. It has been reported that crocetin attenuated TNBS-induced colitis in mice by reducing inflammatory cytokines and lipid peroxidation [13]. A previous study has also proved that crocetin treatment protected against burn-induced intestinal injury via inhibiting oxidative stress and inflammatory response [14]. Additionally, crocetin could inhibit the growth and metastasis of tumor cells both *in vitro* and *in vivo* [15–17]. However, the potential role of crocetin on RIII has not been reported. In this study, we aimed to investigate whether and how crocetin protected against RIII.

## 2. Materials and Methods

### 2.1. Cell Culture

Rat intestinal epithelial IEC-6 cells were obtained from the American Type Culture Collection (ATCC, Manassas, VA, USA). Cells were maintained in high-glucose Dulbecco's modified Eagle's medium (Sigma, St Louis, MO, USA; no. D5796) with 10% fetal bovine serum, 1% penicillin/streptomycin, and 0.1 U/mL recombinant human insulin at 37°C in a humidified atmosphere containing 5% CO_2_. The culture medium was changed every 2 or 3 d. The cells were passaged as they grew to 70~80% confluence, and cells before 20^th^ passages were used for the experiments.

### 2.2. Irradiation and Crocetin Treatment

The radiation procedure was performed according to our previously described protocols [4]. Briefly, IEC-6 cells were exposed to 10 Gy doses of radiation using a linear accelerator (Siemens PRIMUS) at a dose-rate of 300 cGy/min. IEC-6 cells were seeded into 96-well plates at a density of 1 × 10^4^ cells/well and grown to 70%~80% confluence prior to experiment. After 10 Gy radiation, IEC-6 cells were replaced with serum-free DMEM-F12 medium and subsequently treated with different doses of crocetin (0, 0.1, 1, 10, and 100 *μ*M, MP Biomedicals, Santa Ana, CA, USA; CAS no.: 27876-94-4), then incubated for 24 h at 37°C. After 24 h incubation, the culture medium was collected for biochemical assay and ELISA and then replaced with new fresh serum-free medium for subsequent condition of IEC-6 cells. To determine the most effective concentration of crocetin in the following experiments, cell viability was assessed daily for the next 7 days after radiation. Further studies were performed at the most effective concentration to improve cell viability.

### 2.3. Cell Viability Assay

The viability of IEC-6 cells was assessed by CCK-8 assay, and all the steps followed the manufacturer's instruction (Dojindo Laboratories, Kumamoto, Japan; no. CK04). IEC-6 cells were cultured in 96-well plates with a density of 1 × 10^4^ cells/well for 24 h. After 10 Gy radiation and treatment with different doses of crocetin for 24 h, 10 *μ*L of CCK-8 was added to each well and for incubation for another 1 h at 37°C. Cell viability was measured daily for 7 consecutive days after radiation. Absorbance of each well was determined at 450 nm using a Multiskan Spectrum (Thermo Fisher, CA, USA). The experiment was independently repeated at least three times.

### 2.4. Biochemical Measurements

Malondialdehyde (MDA, Beyotime Institute of Biotechnology, Shanghai, China; no. S0131) levels, superoxide dismutase (SOD, Abcam, Cambridge, MA, USA; no. ab65354) activities, catalase (CAT, Sigma, St. Louis, MO, USA; no. CAT100) activities, glutathione peroxidase (GPx, Beyotime Institute of Biotechnology, Shanghai, China; no. S0056) levels, and myeloperoxidase (MPO, Abcam, Cambridge, MA, USA; no. ab105136) activities in the cell culture supernatants were measured at 1, 3, 5, and 7 d after radiation using commercial assay kits, respectively, according to the manufacturer's protocols.

### 2.5. Cell Apoptosis Assay

Cell apoptosis was detected at 1, 3, 5, and 7 days after radiation using Annexin V-FITC/PI Apoptosis Detection Kit (BD Biosciences, San Diego, CA, USA) based on our previously described procedures [18]. Briefly, IEC-6 cells were plated in 6-well plates at a concentration of 1 × 10^5^ cells/well. The cells in all groups were incubated and then harvested at 1, 3, 5, and 7 d after treatment of crocetin, washed with PBS twice, resuspended in binding buffer, and stained with Annexin V and propidium iodide (PI) for 10 min at room temperature in the dark. Annexin V fluorescence was measured using a flow cytometer (BD Biosciences), and the membrane integrity of the cells was simultaneously assessed by the PI exclusion method.

### 2.6. Cytokine Assay

Proinflammatory cytokines tumor necrosis factor-*α* (TNF-*α*, R&D Systems, Minneapolis, MN, USA; no. PMTA00B), interleukin-1*β* (IL-1*β*, RayBiotech, Peachtree Corners, GA, USA; no. ELM-IL1b-1), and interferon-*γ* (IFN-*γ*, R&D Systems, Minneapolis, MN, USA; no. PMIF00) levels were obtained from the cell culture supernatants at 1, 3, 5, and 7 days after radiation and were measured using ELISA kits according to the manufacturer's instructions.

### 2.7. Statistical Analysis

The differences of all measured parameters among groups were analyzed by one-way analysis of variance followed by Student-Newman-Keuls- (SNK-) q test and between two groups by Student *t*-test. All analyses were performed with SPSS statistics package (IBM SPSS, Chicago, IL, USA). Data were considered statistically significant for *P* < 0.05.

## 3. Results

### 3.1. Crocetin Improved the Survival of Irradiated IEC-6 Cells

**Figure 1:**
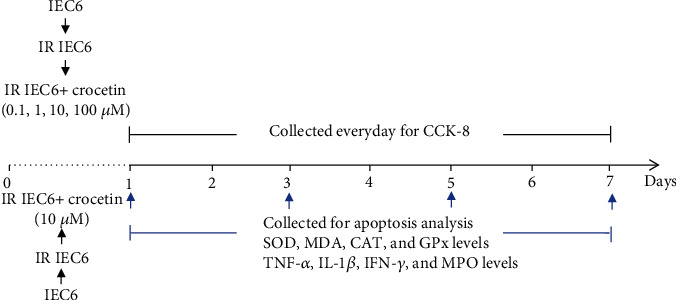
*In vitro* experiment design. IEC-6 cells were exposed to 10Gy of radiation, followed by treatment of different doses of crocetin (0.1 *μ*M, 1 *μ*M, 10 *μ*M, and 100 *μ*M) for 24 h, and culture supernatants were collected for CCK-8 assay from day 1 to day 7 after radiation. Apoptosis was detected on days 1, 3, 5, and 7 after radiation. The levels of SOD, CAT, GPx, MDA, MPO, TNF-*α*, IL-1*β*, and IFN-*γ* in culture supernatants were measured on days 1, 3, 5, and 7 after radiation. SOD: superoxide dismutase; CAT: catalase; GPx: glutathione peroxidase; MDA: malondialdehyde; MPO: myeloperoxidase; TNF-*α*: tumor necrosis factor-*α*, IL-1*β*: interleukin-1*β*, IFN-*γ*: interferon-*γ*; IR: irradiation group.

**Figure 2:**
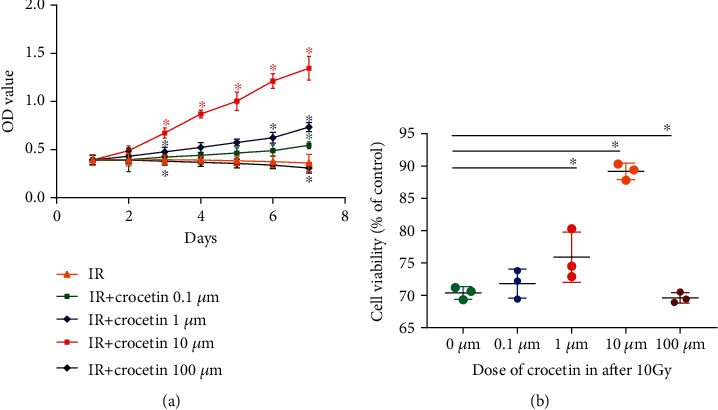
Crocetin improved the survival of irradiated IEC-6 cells in a dose-dependent manner (0.1 *μ*M, 1 *μ*M, and 10 *μ*M). IEC-6 cells were treated with different doses of crocetin (0.1 *μ*M, 1 *μ*M, 10 *μ*M, and 100 *μ*M) after exposure of 10 Gy radiation. (a) Cell viability was detected by CCK-8 from day 1 to day 7 after radiation. (b) Cell viability was detected by CCK-8 on day 3 after radiation. Data represent mean ± SD of three independent experiments. ∗ represents *P* < 0.05 as compared to the IR group. IR group: irradiation group.

To evaluate the therapeutic mechanisms of crocetin in radiation-induced intestinal injury (RIII), we established *in vitro* experimental systems (Figure 1). To determine the optimal concentration of crocetin on irradiated IEC-6 cells, the cell viability of each group was tested by CCK-8 assay. The cell viability of IEC-6 cells was significantly decreased after radiation (Figure 2(b)), whereas treatment with crocetin at concentrations of 0.1 *μ*M, 1 *μ*M, and 10 *μ*M improved the survival of irradiated IEC-6 cells in a dose-dependent manner with the maximal effect achieved at 10 *μ*M (Figures 2(a) and 2(b)). In contrast, 100 *μ*M of crocetin showed a decrease on the cell viability of irradiated IEC-6 cells compared to that of the irradiated group (Figures 2(a) and 2(b)). According to the results, 10 *μ*M was the most effective dose of crocetin to improve the viability of irradiated IEC-6 cells, which was used for subsequent experiments.

### 3.2. Crocetin Attenuated Oxidative Stress in Irradiated IEC-6 Cells

**Figure 3:**
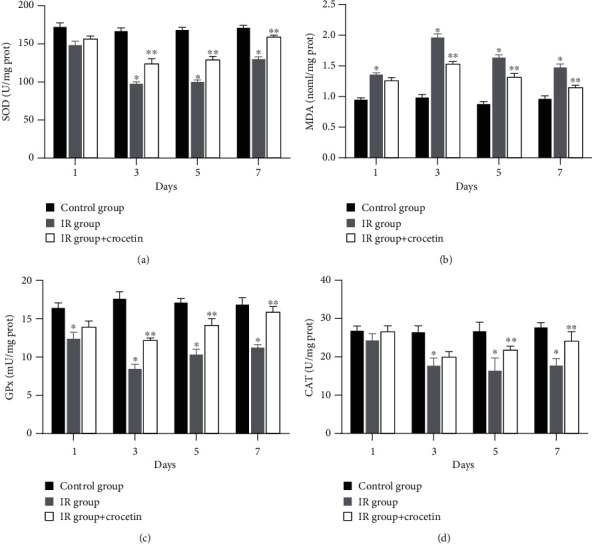
Crocetin attenuated oxidative stress in irradiated IEC-6 cells. The activities of (a) SOD, (b) MDA, (c) GPx, and (d) CAT in cell culture supernatants were detected on days 1, 3, 5, and 7 after radiation. Data were expressed as mean ± SD of three independent experiments. ∗*P* < 0.05 compared to the control group, ∗∗*P* < 0.05 compared to the IR group. SOD: superoxide dismutase; MDA: malondialdehyde; GPx: glutathione peroxidase; CAT: catalase; IR group: irradiation group, IEC-6 cells exposed to 10G y of radiation. Control group: IEC-6 cells without irradiation or crocetin.

To investigate the effect of crocetin on oxidative stress, we examined the levels of SOD, GPx, CAT, and MDA in culture supernatants by colorimetric assays. While radiation led to increased level of MDA, this increase was alleviated by crocetin (Figure 3(b), *P* < 0.05). Conversely, treatment of irradiated IEC-6 cells with crocetin significantly elevated the activities of endogenous antioxidant enzymes (SOD, GPx, and CAT), compared to the irradiated group (Figures 3(a), 3(c), and 3(d), *P* < 0.05). These data suggested that crocetin exerted an antioxidant effect in irradiated IEC-6 cells.

### 3.3. Crocetin Ameliorated Apoptosis in Irradiated IEC-6 Cells

**Figure 4:**
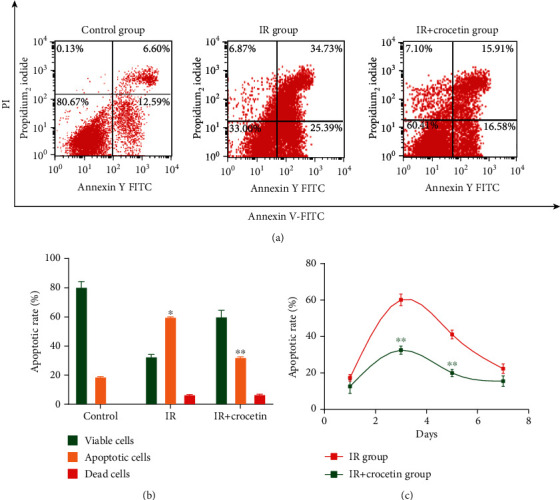
Crocetin ameliorated apoptosis in irradiated IEC-6 cells. (a) Apoptosis of IEC-6 cells was detected by flow cytometry after Annexin V/PI staining 3 days after radiation. The left upper quadrant contains necrotic cells (%); the upper right quadrant contains late apoptotic cells (%); the lower left quadrant contains live cells (%); and the lower right quadrant contains early apoptotic cells (%). (b) The percentage of total apoptotic cells and dead cells was calculated on day 3 after radiation. (c) Apoptotic ratio of IEC-6 cells was detected by Annexin V/PI double staining at 1, 3, 5, and 7 days after radiation. Data were expressed as mean ± SD of three independent experiments. ∗*P* < 0.05 compared to the control group, ∗∗*P* < 0.05 compared to the IR group. IR group: irradiation group, IEC-6 cells exposed to 10 Gy of radiation. Control group: IEC-6 cells without irradiation or crocetin.

We further evaluated the effect of crocetin on apoptosis of irradiated IEC-6 cells by Annexin V/PI double staining. The percentage of apoptotic cells increased after radiation compared to the control group (Figure 4(b), *P* < 0.05), whereas crocetin dramatically reduced the apoptosis of irradiated IEC-6 cells on day 3 and day 5 (*P* < 0.05), with less effects on day 7 (Figures 4(a)–4(c)). These results indicated that crocetin reduced radiation-induced intestinal epithelial apoptosis.

### 3.4. Crocetin Inhibited Inflammation in Irradiated IEC-6 Cells

**Figure 5:**
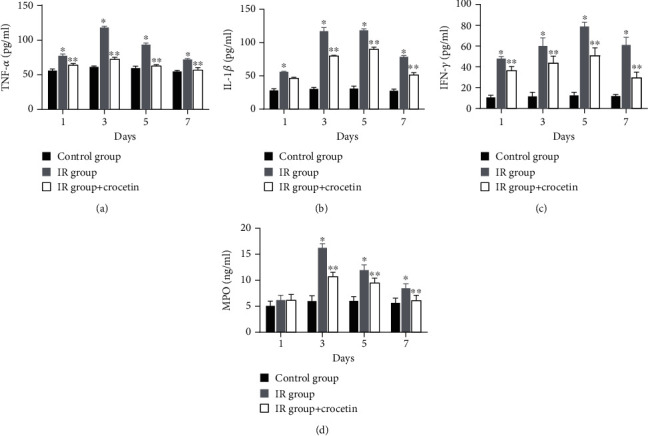
Crocetin inhibited inflammatory response in irradiated IEC-6 cells. The levels of (a) TNF-*α*, (b) IL-1*β*, (c) IFN-*γ*, and (d) MPO in cell culture supernatants were measured on days 1, 3, 5, and 7 after radiation. Data were expressed as mean ± SD of three independent experiments. ∗*P* < 0.05 compared to the control group, ∗∗*P* < 0.05 compared to the IR group. TNF-*α*: tumor necrosis factor-*α*, IL-1*β*: interleukin-1*β*, IFN-*γ*: interferon-*γ*; MPO: myeloperoxidase; IR group: irradiation group, IEC-6 cells exposed to 10 Gy of radiation. Control group: IEC-6 cells without irradiation or crocetin.

To explore the effect of crocetin on inflammatory response in irradiated IEC-6 cells, the levels of proinflammatory cytokines in culture supernatants were assessed. Exposure to radiation remarkably increased the levels of TNF-*α*, IL-1*β*, and IFN-*γ*, while administration of crocetin dramatically decreased this effect (Figures 5(a)–5(c), *P* < 0.05). Consistent with the results of proinflammatory cytokines, crocetin significantly suppressed MPO activity (Figure 5(d), *P* < 0.05), suggesting crocetin attenuated radiation-induced inflammation in IEC-6 cells.

## 4. Discussion

Though agents ameliorating radiation-induced damage by reducing oxidants stress and inflammation may exert protective effects against RIII, the potential toxicity and tumorigenicity must be addressed before their clinical application [6]. In contrast, crocetin could be an alternative radioprotector for RIII with low toxicity [19, 20] and antitumor properties [11, 21]. In our study, we demonstrated the protective effects of crocetin against radiation-induced injury in intestinal epithelial cells and the underlying mechanisms could be attributed to inhibition of oxidative stress, cellular apoptosis, and inflammatory response, suggesting a safe and effective strategy for RIII.

There are some important discoveries in our work. First, the protective effects of crocetin in different concentrations on irradiated IEC-6 cells were investigated. In this study, we demonstrated that lower concentrations (0.1 *μ*M, 1 *μ*M, and 10 *μ*M) of crocetin improved the survival of irradiated IEC-6 cells in a dose-dependent manner, showing the most pronounced effect at the dose of 10 *μ*M. Consistent with our findings, Yoshino et al. found that crocetin at 1~10 *μ*M protected HT22 cells against A*β*_1-42_-induced neuronal cell death [22]. Conversely, it was reported previously that high doses of crocetin exerted cytotoxic effects on healthy monocytes and Alzheimer's disease monocytes [23]. Our study also found that 100 *μ*M of crocetin decreased cell viability whereas no cytotoxicity was observed at 0.1~10 *μ*M, suggesting the safe concentration of crocetin should be lower than 100 *μ*M. These findings suggested that 10 *μ*M was relatively a safe and effective dose of crocetin to protect irradiated IEC-6 cells.

Second, our study demonstrated the mechanisms of crocetin on RIII. Previous studies showed that crocetin exerted beneficial effects on tissue regeneration by reducing oxidative stress, inhibiting cellular apoptosis, and attenuating inflammatory response [24–26]. Recently, a study further investigated that crocetin protected ultraviolet A radiation-induced skin damage by reducing oxygen species production and cellular apoptosis [27]. Similar with these studies, we observed that crocetin inhibited oxidative stress, the occurrence of apoptosis, and inflammation in irradiated IEC-6 cells, suggesting crocetin could attenuate intestinal toxicity induced by radiation.

Though some substances have shown variable degrees of radioprotective properties, the application of most agents is hindered by toxicity and narrow therapeutic time windows [28]. Crocetin has been reported to treat a wide range of diseases with low toxicity [19, 20]. Milajerdi et al. suggested that LD_50_ values of saffron stigma extracts containing crocetin could be very higher than the therapeutic dose [29]. A clinical study also reported that no adverse changes in volunteers were observed after crocetin was administrated at the dose of 37.5 mg/d for 4 weeks [30]. Moreover, crocetin could inhibit the proliferation and invasion of various tumor cells including intestinal cancer [31]. Kim et al. have demonstrated that crocetin could increase the death of HCT-116 colorectal cancer cells [11]. Ray et al. have also demonstrated that crocetin could induce p53-mediated cell death by p73-mediated FAS-FADD-caspase-8 activation and BID cleavage in colorectal cancer cells [32]. As RIII commonly occurs in patients with abdominopelvic malignancies receiving local radiation therapy, crocetin represents a promising therapy to attenuate radiation-induced injury of intestine and, at the same time, inhibit tumor growth. However, the possible optimal doses in vivo still need to be further studied.

This study has potential limitations. First, the effect of crocetin on tumor cell lines after radiation was not studied because previous studies have reported the antitumor effects of crocetin on various tumor cells [11, 31]. Second, the solubility and the bioavailability of crocetin require optimization before being used as an effective radioprotective agent [12]. This problem may be solved with cyclodextrins or similar molecules. For example, Wong et al. suggested that crocetin-*γ*-cyclodextrin inclusion complex could enhance the solubility, bioavailability, and applicability of crocetin [33]. Puglia et al. showed that solid lipid nanoparticles containing crocetin improved its solubility, stability, and pharmacokinetic properties, offering an appropriated approach to resolve this issue [34]. Third, this is a study in cell model only providing preclinical clues for radioprotection of crocetin; more studies are needed to demonstrate its effects on RIII in animal models and further in clinical studies.

## 5. Conclusions

In conclusion, the present study suggests that crocetin could be an attractive agent for RIII not only attenuating intestinal injury induced by radiation via inhibiting oxidative stress, cellular apoptosis, and inflammatory response but also improving the efficacy of cancer cure with potential antitumor effects.

## Data Availability

The data used to support the findings of this study are available from the corresponding author upon reasonable request.
